# Effect of Manganese Distribution on Sensor Properties of SnO_2_/MnO_x_ Nanocomposites

**DOI:** 10.3390/nano13091437

**Published:** 2023-04-22

**Authors:** Rodion Eshmakov, Darya Filatova, Elizaveta Konstantinova, Marina Rumyantseva

**Affiliations:** 1 Chemistry Department, Moscow State University, 119991 Moscow, Russia; 2 Physics Department, Moscow State University, 119991 Moscow, Russia

**Keywords:** nanocrystalline tin oxide, thermally activated gas sensor, nitrogen oxide, carbon monoxide, microstructure effect, manganese doping, surface modification

## Abstract

Nanocomposites SnO_2_/MnO_x_ with various manganese content (up to [Mn]/[Sn] = 10 mol. %) and different manganese distribution were prepared by wet chemical technique and characterized by X-ray diffraction, scanning electron microscopy (SEM) with energy dispersive X-ray (EDX) analysis and mapping, IR and Raman spectroscopy, total reflection X-ray fluorescence, mass-spectrometry with inductive-coupled plasma (ICP-MS), X-ray photoelectron spectroscopy (XPS), electron paramagnetic resonance (EPR) spectroscopy. A different distribution of manganese between the volume and the surface of the SnO_2_ crystallites was revealed depending on the total Mn concentration. Furthermore, the identification of surface MnO_2_ segregation was performed via Raman spectroscopy. There is a strong dependence of the sensor signal toward CO and, especially, NO) on the presence of MnO_2_ surface segregation. However, manganese ions intruding the SnO_2_ crystal structure were shown to not almost effect on sensor properties of the material.

## 1. Introduction

SnO_2_ is a well-known material for resistive-type semiconductor gas sensors [1]. It is known that an increase in the SnO_2_ selectivity and sensor response toward various gases can be achieved by surface modification with catalytic oxides to obtain a composite [2]. Therefore, manganese oxides (MnO_2_, Mn_2_O_3_), which have high catalytic activity in redox reactions involving oxygen and its compounds [3,4], are of interest for use as SnO_2_ modifiers [5,6,7]. Among many methods of synthesis of SnO_2_-based materials, one of the simplest and easy to use are various modifications of the sol-gel technique [8,9,10], which provides materials with high specific surface area [11,12] due to small grain size and allows to use different ways of introducing a modifier to the blank SnO_2_ [13,14]. To prepare nanocomposites with SnO_2_ surface modified with manganese oxides but not bulk doping, it is logical to use the appropriate modification procedures, such as impregnation by a solution of manganese-containing compound with following annealing on air to decompose manganese precursor to oxide [15]. The choice of Mn-containing precursor is based on its thermal properties, solubility, and storage stability: many manganese salts [7,16] seem to be suitable as well as soluble in organic solvents manganese (III) acetylacetonate [17,18].

So, different ways of preparing SnO_2_/MnO_x_ materials were suggested. At the same time, the structure and sensor properties of SnO_2_/MnO_x_ composites have not been adequately studied. There are also controversial and arguable data characterizing such composites’ phase composition and manganese oxidation state. For example, SnO_2_/MnO_x_ materials were prepared in the same manner in the works [8,11,12], but the ascribed oxidation state of manganese is different in all these articles and varies from +2 to +4. A very questionable conclusion about Mn^2+^ introduction in SnO_2_ lattice was made [8] in contradiction with known X-ray diffraction data: the observed lattice volume decreasing can’t be a consequence of the effect of Sn (IV) substitution by Mn (II) because of larger ionic radius of the latter one [19,20,21]. However, an exciting study of Mn_2_O_3_ solubility in SnO_2_ at 600 °C was performed [11], but these data were related to high Mn: Sn ratios. It seems the direct analysis of the chemical state of manganese in materials with low Mn content (less than dozens molar percent) is a complicated task, and numerous authors had a failure using traditional methods such as X-ray photoelectron spectroscopy [6,19].

The main aim of this work is to clarify microstructure parameters, the chemical state of elements and their distribution in materials based on sol-gel synthesized SnO_2_, which was modified by impregnation-annealing (at 300 °C) technique with MnO_x_ using manganese(III) acetylacetonate as a manganese-containing precursor, with several contemporary analytical techniques (total reflection X-ray fluorescence, Raman, electron paramagnetic resonance, X-ray photoelectron spectroscopies, mass-spectroscopy with inductive-coupled plasma, etc.). Furthermore, such data are necessary to elucidate sensor properties of materials which were studied when detecting model gases CO and NO in dry air to reveal the role of manganese in sensor signal formation.

## 2. Materials and Methods

### 2.1. Materials Synthesis

#### 2.1.1. Synthesis of Nanocrystalline SnO_2_

Nanocrystalline SnO_2_ was prepared by α-stannic acid precipitation from tin (IV) chloride aqueous solution with concentrated ammonia solution. A solution of NH_3_·H_2_O (13 M) was added dropwise into the water solution of SnCl_4_·5H_2_O (16.3 g, 0.3 M) with vigorous stirring until pH ~6 was reached. The synthesis was carried out at room temperature (RT). As a result, the following reaction took place:

(1) $${\mathrm{SnCl}}_{4}+4{\mathrm{NH}}_{3}\cdot H_{2}O+\left(n-2\right)H_{2}O\to {\mathrm{SnO}}_{2}\cdot {\mathrm{nH}}_{2}O↓+4{\mathrm{NH}}_{4}\mathrm{Cl}$$

The white dense gel-like precipitate was separated by centrifugation. Then it was washed ten times with 0.1 M NH_4_NO_3_ solution (to prevent peptization) and with distilled water until chloride ions were removed. An opalescence absence was verified by adding AgNO_3_ solution (0.01 M). Next, the SnO_2_·nH_2_O gel was dried at 50 °C for 24 h. The resulting glassy solid (6.8 g, yield 87%) was grounded to a powder state in an agate mortar and annealed in air at 300 °C for 24 h (sample name–SnO2_300, Table 1).

#### 2.1.2. Synthesis of SnO_2_/MnO_x_ Nanocomposites

The «impregnation and annealing» method obtained SnO_2_/MnO_x_ nanocomposite as powders. Manganese (III) acetylacetonate (Mn(acac)_3_) used in the synthesis was prepared by a well-known procedure described in [22] and recrystallized by slow evaporation on air at RT from saturated solution in chloroform with the addition of ×5 excess of heptane. The mushy mixtures of SnO_2_ nanocrystalline powder and fresh manganese (III) acetylacetonate ethanolic solution were taken in Mn:Sn = 1:99 (sample name–SnO2_Mn1) and 10:90 (sample name–SnO2_Mn10) molar proportions, were dried at 50 °C and annealed at 300° C for 24 h. 

Both samples were treated with hydrochloric and oxalic acids to remove manganese-containing segregation from the tin (IV) oxide surface. Weighed amounts (100 mg) of SnO2_Mn1 and SnO2_Mn10 were deposited into small test tubes, and 1.00 mL of saturated (at RT) H_2_C_2_O_4_ solution was added to each probe. Prepared suspensions were heated at 70 °C in an ultrasonic bath for 1 h. After that, 0.100 mL of HCl (conc.) was mixed with suspensions, and they were left for one night. Finally, precipitates were separated by centrifugation, washed with distilled water five times, and dried at 50 °C (sample names–SnO2_Mn1_w, SnO2_Mn10_w).

### 2.2. Materials Characterization

The thermal stability of manganese (III) acetylacetonate used for materials preparation was studied by thermogravimetry (TG) combined with differential scanning calorimetry (DSC) and mass-spectrometry (MS) using NETZSCH STA 409 PC thermobalance with NETZSCH QMS 403C MS system (NETZSCH-Gerätebau GmbH, Selb, Germany). Samples were heated in a corundum crucible from RT to 550 °C (5 °C/min) in the airflow (30 mL/min).

Fourier transforms infrared (FTIR) spectroscopy measurements were performed to check the completeness of Mn(acac)_3_ thermolysis on Frontier (Perkin Elmer Inc., Beaconsfield, UK) spectrometer in the transmission mode in the wavenumber range of 400–4000 cm^−1^ with a step of 1 cm^−1^. Samples (about 5 mg) were ground with 100 mg of potassium bromide (Aldrich, for FTIR analysis) and pressed into tablets 6 mm in diameter.

The Microstructure and morphology of prepared materials were investigated by scanning electron microscopy (SEM) with Zeiss Supra 40 FE-SEM microscope (Carl Zeiss, Inc., Oberkochen, Germany) with an in-lens detector. The accelerating voltage was set to 10 kV, and the aperture size was 30 microns.

The specific surface area (S_surf_) of nanocrystalline oxides was measured by low-temperature nitrogen adsorption using a Chemisorb 2750 instrument (Micromeritics, Norcross, GA, USA). The surface area is available for adsorption was calculated using the BET model (Brunauer, Emmett, Teller). The size of aggregates whose surface is available for gas adsorption (d_BET_) was calculated for each material in the approach of uniform spherical shape:

(2) $$d_{\mathrm{BET}}=\frac{6}{{\rho S}_{\mathrm{surf}}}$$

where ρ is the density of material (accepted equal to the blank SnO_2_—6.95 g/cm^3^).

The phase composition of the obtained materials was investigated by X-ray diffraction (XRD) with a DRON-4-07 (Burevestnik, St. Petersburg, Russia) and Rigaku D/MAX 2500 (Rigaku, Japan, Tokyo) diffractometers equipped with CuKα (λ = 1.5406 Ǻ) radiation source. Obtained diffraction patterns were processed using the STOE WinXPOW software (v. 1.06). The ICDD PDF2 database was used to identify the crystal phases. The SnO_2_ crystalline grain size (d_XRD_) was calculated using the Scherrer formula for (110), (101) and (111) diffraction maxima.

To clarify the manganese state in SnO_2_/MnO_x_ materials, the phase composition and crystal structure of the synthesized samples were also studied by Raman spectroscopy. The studies were carried out without specific sample preparation on the i-Raman Plus spectrometer (B&W Tek, Plainsboro, NJ, USA) equipped with a BAC 151C microscope and 532 nm laser and Horiba LabRam Evolution Raman spectrometer (Horiba Ltd., Minami-ku, Kyoto, Japan) equipped with a single-mode CW-laser (633 nm, 170 mW) in the confocal microscope (Olympus, Shinjuku, Tokyo, Japan) scheme with a spatial amplification of ×50 and numeric aperture 0.25. During the measurements, only 25% of the maximum possible laser power was used. Spectra processing (baseline correction, peak fitting, and integration) was implemented with Origin Pro 2021 v. 9.8.0.200 software package.

Elemental composition mapping was performed using NVision 40 microscope (Carl Zeiss, Oberkochen, Germany) with an energy dispersive X-ray analyzer (EDX) X-MAX (Oxford Instruments, Abingdon, Oxfordshire, UK) at an accelerating voltage of 20 kV and aperture of 60 microns.

Total reflection X-ray fluorescence analysis (TXRF) was used to determine the elemental composition of SnO2_Mn1 and SnO2_Mn10. Sample SnO2-300 was taken as a control. The analysis was carried out with Bruker S2 Picofox TXRF spectrometer (Bruker Corporation, Billerica, MA, USA). Special techniques of sample preparation were used. To determine the total manganese content, the sample (6 mg) was dispersed in 0.100 mL of polyvinyl alcohol 0.3 g/L solution, 0.300 mL deionized water and 0.100 mL standard Co^2+^ solution with a concentration of 100 mg/L. A 0.005 mL probe of stirred suspension was taken, placed on a quartz support and dried before the measurements. An attempt to study the manganese distribution between the SnO_2_ surface and lattice was also made: first, weighed material (12 mg) was treated with 1.00 mL of saturated (at RT) oxalic acid solution in an ultrasonic bath for 1 h at 70 °C with followed addition of 0.100 mL of HCl (conc.) and kept for one night at RT. Second, the 0.400 mL probe was taken from the supernatant solution, and 0.100 mL of standard (Co^2+^ solution, 100 mg/L) was added to the specimen. A dried 0.005 mL droplet of this mixture was used to determine the amount of manganese in the MnO_x_ segregation state.

The remaining manganese content in SnO2_Mn1_w and SnO2_Mn10_w samples (which appeared to contain manganese only in the SnO_2_ crystal structure) was studied by inductively coupled plasma mass spectrometry (ICP MS) on a quadrupole ICP mass spectrometer (Agilent 7500c: Agilent Technologies, Santa Clara, CA, USA), which was controlled with a PC using the ChemStation (version G1834B) software package (Agilent Technologies, Santa Clara, CA, USA). Sample SnO2-300 was taken as a blank. Measurements were performed for the ^55^Mn isotope. Before analysis, pre-weighed powders (10 mg) were entirely dissolved in the mixture of HF (conc.) and HCl (conc.) at a ratio of 1:2, respectively, using a two-steps program in closed-type microwave system (Milestone ETHOS Advanced Microwave Labstation, Milestone Srl, Sorisole (BG), Italy) with temperature and pressure control options. The working frequency of the system was 2455 MHz, and the radiated power was 800 W. In the first stage, samples were heated from room temperature to 200 °C for 30 min. Then in the second stage, the reached temperature was kept for 30 min else. The resulting solutions were diluted to a volume of 6 mL by adding deionized water. After that, the 0.100 mL sample probes were diluted 100 times with deionized water. The ICP MS single element standards (Mn) were prepared from the standard solution (High-Purity Standards, Charleston, SC, USA) with a 10 mg/L concentration. The solution of the control sample was used to measure the background signal.

The chemical state of elements (Sn, O, Mn) in SnO2-300 and SnO2_Mn1 was studied by X-ray photoelectron spectroscopy (XPS) using Omicron ESCA+ (Scienta Omicron, Uppsala, Sweden) spectrometer with monochromatized aluminum anode Al K_α_ (1486.6 eV) with neutralizer. The binding energy step was 0.1 eV/s. Transmittance energy was 20 eV. Spectra of C 1s (trace carbon), O 1s, Sn 3d, Mn 3s, and Mn 2p were recorded. Unifit v. 2006 was used for spectra processing by approximating Gaussian and Lorentzian peak functions combination and background fitting. No special sample preparation procedure was used before analysis.

Oxidative active centers on the surface of prepared materials were studied by thermo-programmed reduction with hydrogen (TPR-H_2_) on Chemisorb 2750 instrument (Micromeritics, Norcross, GA, USA) without specific sample preparation. Pre-weighed specimen (15–20 mg) was placed into a quartz flow test tube equipped with a thermocouple located in close proximity to the sample. The flow value 50 mL/min of Ar-H_2_ mixture (8% vol. H_2_) was set, and the temperature was raised by 10 °C/min till 800 °C. Hydrogen consumption during sample reduction was detected by a thermal conductivity detector (TCD) and recorded in arbitrary units. The volume of consumed hydrogen (V_H2_) was calculated by the following equation:

(3) $$V_{H2}=k\times \int_{T1}^{T2}\frac{\mathrm{TCD}}{m}\cdot \mathrm{dT}$$

The proportion quotient k = 0.28 mL/(g·a.u.) was determined by calibration measurements of a standard Ag_2_O sample with H_2_ absorption of 96.55 mL/g at normal conditions. The ideal gas approximation was used to recalculate the amount of consumed H_2_ at real temperature and pressure.

Paramagnetic centers on the surface of the synthesized materials were investigated by electron paramagnetic resonance spectroscopy (EPR) with Bruker ELEXSYS-580 spectrometer (Bruker Corporation, Billerica, MA, USA) at working frequency 9.5 GHz and apparatus sensitivity 5·10^10^ spin/Gs. A standard sample with Mn^2+^ ions was used to determine the g-factor values. A reference sample of CuCl_2_·2H_2_O was used to calculate the concentrations of paramagnetic centers in the material. Theoretical spectra simulated in Easyspin software were used to prove the material’s qualitative and quantitative composition of spin centers. Experimental spectra were obtained at 298 K. Samples were analysed and diluted 100 times with blank SnO_2_ (SnO2-300).

Gas sensor properties of prepared materials (excepting SnO2_Mn10_w) toward CO and NO were investigated by measuring the resistance of thick films in situ in a flow cell under a controlled gas flow of 100 ± 0.1 mL/min. The gas mixture for measurements was prepared by dilution of certified gas mixtures (CO 2530 ppm in N_2_, NO 246 ppm in N_2_) with dry synthetic air (relative humidity RH about 1%) using a pure air generator (GChV-1,2-3,5; Chimelectronika, Moscow, Russia) and RRG-12 electron mass-flow controllers (Eltochpribor, Zelenograd, Moscow, Russia). The concentrations of NO in gas mixtures were additionally verified with a Teledyne API N500 CAPS NOX Analyzer (Teledyne API, Inc., San Diego, CA, USA). The sensor signal values were calculated as follows:

(4) $$S=\frac{R_{\mathrm{air}}-R_{\mathrm{gas}}}{R_{\mathrm{gas}}}$$

where R_air_ is sample resistance in pure air, and R_gas_ is sample resistance in air, containing a preassigned concentration of target gas.

Sensitive layers were deposited on chips with platinum microheaters in the form of mushy dispersion in α-terpineol, which was evaporated into the air by heating the layer at 300 °C for 1 h. Heating control and resistance values reading were carried out automatically by a 4-channel analyzer with a measuring range from 1 to 10^12^ Ω at 1.3 V. Temperature and concentration signal dependencies were investigated. In the first case test gas mixture with constant concentration (CO 20 ppm; NO 4 ppm) was supplied to the flow cell alternatively with air (exposure time 15 min) at a fixed temperature for 1.5 h. The temperature was varied with a 30 °C step in the 30–300 °C. In the second case, the data were recorded at the constant pre-determined temperature, corresponding to maximum and stable sensor signal under various test gas concentrations (5, 10, 20, 50 and 100 ppm at 270 °C for CO; 0.5, 1, 2, 4 and 8 ppm at 270 °C for NO).

## 3. Results and Discussion

### 3.1. Suitability Test of Manganese(III) Acetylacetonate as a Source of MnO_x_ Modificator for SnO_2_

According to the known crystallographic data, [23] synthesized manganese (III) acetylacetonate (Mn(acac)_3_) adopts δ-Mn(acac)_3_ structure, also named in Inorganic crystal structures database (ICSD) ACACMN23 as it illustrated on Figure S1 (Supplementary Information).

**Figure 1:**
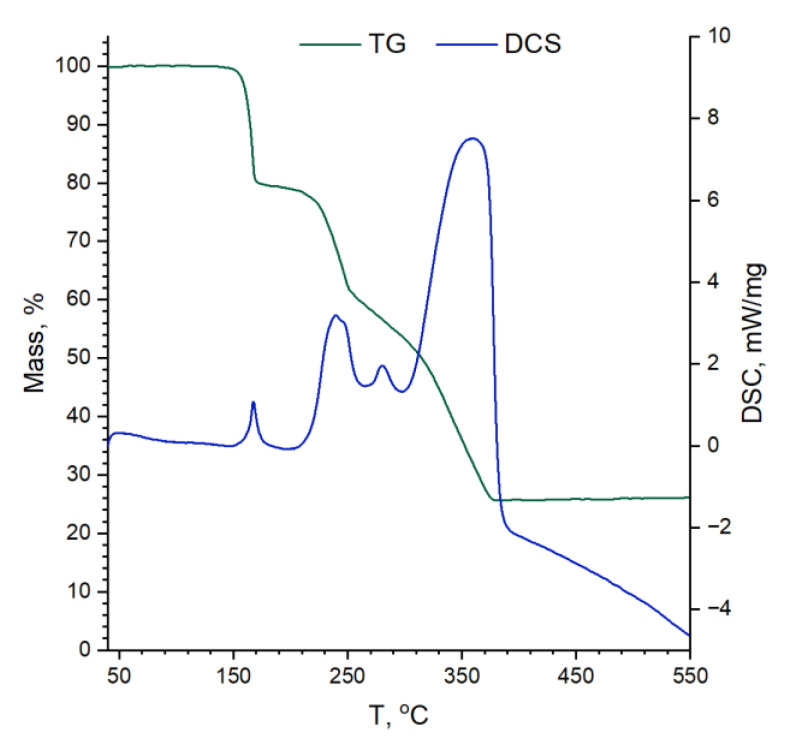
Thermogravimetry and differential scanning calorimetry curves of Mn(acac)_3_ decomposing in airflow.

Thermogravimetry (TG) study jointed with differential scanning calorimetry (DCS) and mass-spectral (MS) detection of volatile decomposition products has shown that decomposition of Mn(acac)_3_ undergoes in 4 stages (Figure 1). Initially, about 20% of sample mass was lost rapidly in the temperature range of 140–160 °C that could be accounted for by one acetylacetonate-ligand loss and substituting it by oxo- or hydroxylic groups. During the next two slower stages (200–380 °C), nearly 75% of the weight was lost due to the formation of MnO_2_ mixed with the remaining carbon. Finally, in the last stage, it is slowly oxidized by air, which is proved by the appearance of a CO_2_^+^ (*m*/*z* = 44 g/mol) signal at a temperature above 380 °C. As demonstrated by DCS data, stages 1–3 are exothermic, and the latter one is endothermic.

**Figure 2:**
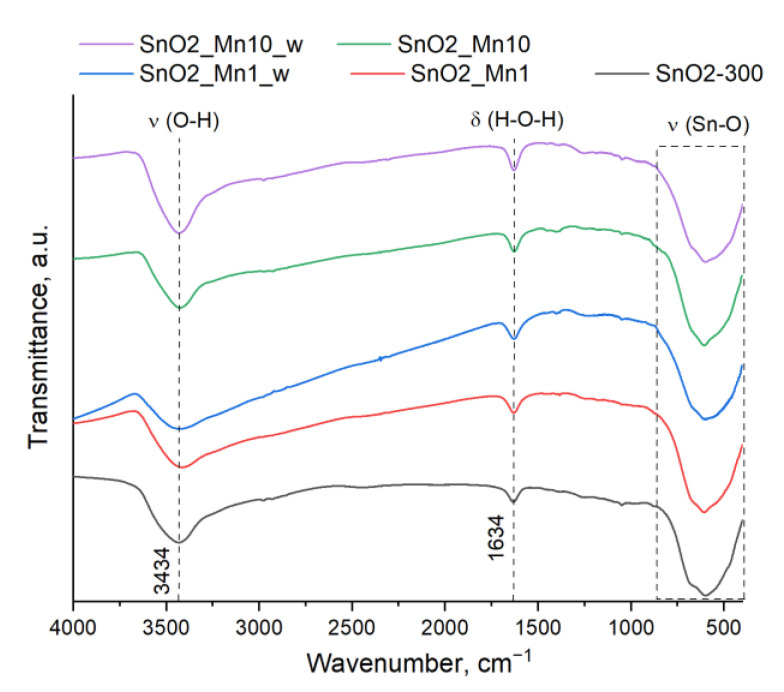
FTIR spectra of blank SnO_2_ and SnO_2_/MnO_x_ materials.

Fourier transforms infrared (FTIR) spectra of blank SnO_2_ and SnO_2_/MnO_x_ powders (Figure 2) show the absence of characteristic bands for C-H-O organic compounds. The only strong absorption bands presented on these spectra correspond to the crystal structure Sn-O vibrations (400–870 cm^−1^), adsorbed water (1634 cm^−1^) and hydroxyl groups (3000–3670 cm^−1^) [24]. So, one can accept that Mn(acac)_3_ decomposes completely during the 24 h annealing of SnO_2_ impregnated with manganese (III) acetylacetonate.

### 3.2. Morphology, Microstructure and Phase Composition of Prepared Materials

**Figure 3:**
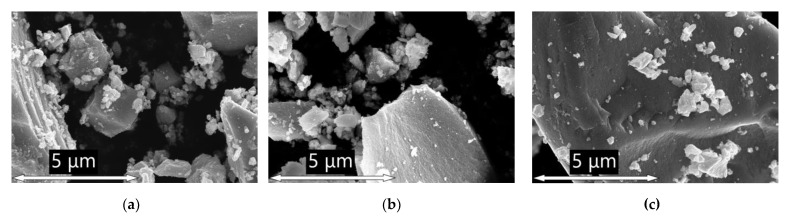
SEM images obtained with Zeiss Supra 40 FE-SEM microscope at ×30·10^3^ magnification of the following samples: (**a**) SnO2-300 (**b**) SnO2_Mn10; (**c**) SnO2_Mn10_w.

Scanning electron microscopy (SEM) has shown that the prepared materials consist of aggregates formed by irregularly shaped particles of various sizes (Figure 3a). Adding manganese (Mn: Sn = 1–10 mol.%) by impregnation-annealing does not lead to visible changes in the morphology of SnO2-300-based materials (Figure 3b). However, the surface of the particles of the etched samples SnO2-Mn1_w and SnO2_Mn10_w seems to be smoother and clearer (Figure 3c).

Although the resolving power of used equipment is insufficient to get sharp images of individual crystalline grains (Figure S2, Supplementary Information), their size (d_SEM_) could be estimated approximately in the 5–10 nm range for each prepared material.

**Figure 4:**
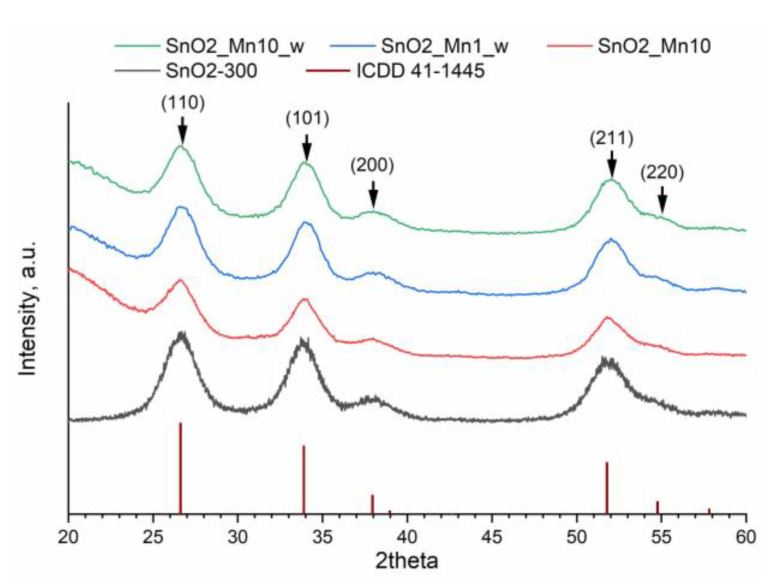
Powder X-ray diffraction patterns of prepared materials compared with a calculated pattern of cassiterite-type SnO_2_ (ICDD 41-1445).

As shown by the X-ray diffraction (XRD) study, synthesized tin (IV) oxide is single phase and matches cassiterite (rutile-type) structure (ICDD 41-1445). As for SnO_2_/MnO_x_ composites, no new crystalline phases were found even in the SnO2_Mn10 sample (Figure 4), which agrees with known data [11]. The size of the coherent scattering region (crystalline grain size, d_XRD_) calculated by the Scherer equation is 3–4 nm for SnO2-300 and slightly larger for SnO2_Mn10 (4.5–5 nm) and etched materials (4–4.5 nm).

Specific surface area (S_surf_) values of all the SnO_2_/MnO_x_ composites are close to each other and equal to 100–105 m^2^/g, slightly less than the S_surf_ of blank SnO_2_ (120 m^2^/g). Therefore, the size of aggregates whose surface is available for gas adsorption (d_BET_) calculated by Formula (2) is about 8 nm for SnO_2_/MnO_x_ materials and near 7 nm for blank SnO_2_ that does not contradict with XRD data and testifies to the gas permeability of observed agglomerates.

The whole information discussed in microstructure and phase composition is summarized in Table 2.

One can see a tendency to increase the SnO_2_ grain size with increased manganese content in the composites. The etched samples SnO2_Mn1_w and SnO2_Mn10_w retain a larger crystallite size than the SnO2-300. So, it could be stated that manganese forces the growth and aggregation of crystallites during the annealing stage of composites preparation.

### 3.3. Elemental Composition and Manganese Surface-Bulk Distribution in SnO_2_/MnO_x_ Materials

#### 3.3.1. Investigation of Surface Composition and Chemical State via Raman Spectroscopy

Raman spectroscopy is more than just a method of phase analysis in the case of studying such materials as SnO_2_/MnO_x_ nanocomposites. The form of the spectrum also depends on the crystallite size and phase distribution in the sample [12,13]. Prepared manganese-containing materials are brown colored, so they absorb visible light significantly, especially in short-wavelength regions. Thus, during the Raman spectra measurements, the excitation radiation will be absorbed quickly in the surface layer of the sample without deep penetration through the sample. Vice versa X-ray radiation during XRD studies has negligible absorbance in the sample and interacts with the whole sample. That’s why Raman spectroscopy will primarily characterize the surface of the sample.

**Figure 5:**
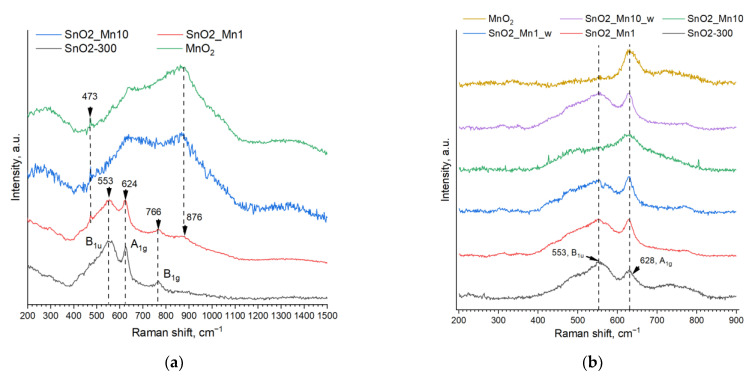
(**a**) Raman spectra of blank SnO_2_, SnO_2_/MnO_x_ nanocomposites with molar Mn:Sn ratio of 1% and 10% and MnO_2_ («pure», «Reakhim») obtained with 532 nm radiation. Intensities were normalized; (**b**) Raman spectra of synthesized materials were obtained with 633 nm radiation. Intensities were normalized. Baseline correction was implemented. The assignment of the frequencies of the SnO_2_ peaks was performed according to the data from [25].

However, strong absorption of laser radiation causes such problems as a lack of Raman signal and sample overheating, which leads to a shift of Raman bands and an increase in background signal. When using a 532 nm laser as a radiation source, the SnO2_Mn10 sample and reference manganese dioxide spectra were uninformative because of the abovementioned problems. However, high-quality spectra were obtained for whiter samples with lower manganese content (Figure 5a).

The spectrum of the SnO2-300 sample corresponds to the spectrum of tin (IV) oxide with the same d_XRD_ grain size described in [25]. Almost the same peaks are found in the spectrum of SnO2_Mn1. Besides, on the spectrum of SnO2_Mn1 there is a well-discernible peak at 473 cm^−1^, which is also present on SnO2_Mn10 and MnO_2_ spectra but almost indistinguishable on the spectrum of blank SnO_2_. There are no SnO_2_ bands in the SnO2_Mn10 spectrum. The MnO_2_ spectrum is precisely the same but contains a wide halo in the range of 500–1200 cm^−1^, which is also present to some extent in the spectrum of SnO2_Mn 1 but is not detected in the spectrum of SnO2-300. An arguable but expected (due to thermal stability data of manganese oxides) conclusion can be made based on these observations: manganese is present as manganese dioxide on the surface of SnO2_Mn1 and SnO2_Mn10 nanocomposites. However, the obtained MnO_2_ spectrum does not correspond to any known MnO_2_ spectrum [26,27].

An attempt was made to override the issues found using a red 633 nm laser instead of a green 532 nm laser as a radiation source. It is supposed to be less absorbed by manganese-containing samples causing overheating to a small extent.

As it turned out, the non-artifact spectra of even the dark manganese-containing samples and MnO_2_ can be acquired using a 633 nm laser (Figure 5b). Moreover, obtained MnO_2_ spectrum matches well with previously published data for pyrolusite-type manganese dioxide [28]. On the contrary, the blank tin (IV) oxide spectrum becomes less informative: the absence of the B_1g_ band and the decreasing intensity of A_1_g are notable.

**Figure 6:**
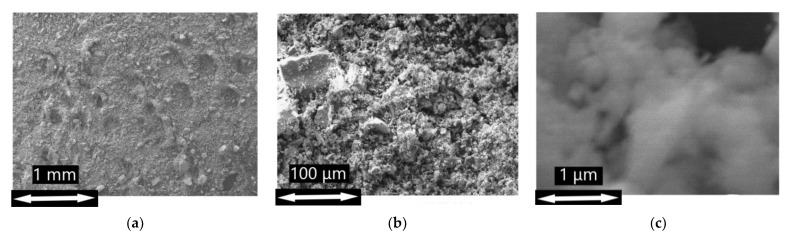

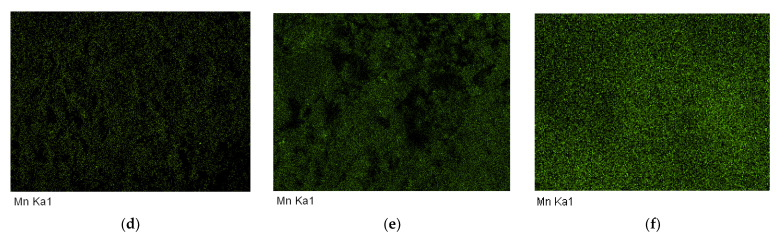
SEM images of SnO2_Mn10 sample at different magnifications: (**a**) ×10^2^; (**b**) ×10^3^; (**c**) ×10^5^; and manganese distribution EDX maps of SnO2_Mn10 sample at same magnifications: (**d**) ×10^2^; (**e**) ×10^3^; (**f**) ×10^5^.

The spectra of SnO2_Mn1, SnO2_Mn1_w and SnO2_Mn10_w presented in Figure 6b are quite similar to the SnO2-300 spectrum in terms of intensity and width of the peaks. Contrary, the SnO2_Mn10 spectrum looks like a combination of MnO_2_ and SnO_2_ spectra. Peaks fitting by Gaussian functions were carried out to prove these diversities quantitatively (Table 3).

Undoubtedly, the SnO_2_ A_1g_ peak (named further as «sharp peak») brings the main contribution to the sharp Raman peak in the spectra of SnO2_Mn1, SnO2_Mn1_w and SnO2_Mn10_w samples, because square ratios of sharp peak and broad peak (designation for a band at 553 cm^−1^) are very similar to blank SnO_2_. Moreover, the color of the sample correlates with the relative intensity of the sharp peak, as shown by I/FWHM values. Likewise, MnO_2_ peak contribution defines the shape of a sharp peak in the SnO2_Mn10 spectrum.

In conclusion, it is very reasonable to consider the presence of MnO_2_ segregation on the surface of SnO_2_/MnO_x_ nanocomposites SnO2_Mn1 and SnO2_Mn10. Concerning the etched samples SnO2_Mn1_w and SnO2_Mn10w, it is difficult to imagine the existence of MnO_2_ on their surface after the treatment by concentrated H_2_C_2_O_4_/HCl mixture at elevated temperature, but their color indicates the presence of manganese in the material.

#### 3.3.2. Direct Determination of Manganese Concentration and Distribution

Although Raman spectroscopy mainly detects MnO_2_ segregation on the SnO_2_ surface in SnO_2_/MnO_x_ nanocomposites, this method is not applicable for determining the uniformity of the manganese distribution over the surface and establishing the quantitative composition. That is why energy dispersive X-ray analysis (EDX) and elemental composition mapping for Sn, Mn, O and trace carbon were carried out for SnO2_Mn1, SnO2_Mn10 and etched samples SnO2_Mn10_w at ×10^2^, ×10^3^ and ×10^5^ magnification as the manganese in SnO2_Mn10 was suspected to be distributed unevenly. However, performed mapping at the chosen scales proves the uniform surface distribution of manganese in prepared materials, even in the SnO2_Mn10 (Figure 6 and Figure S3, Supplementary Information).

The interesting detail is the detection of manganese in the SnO2_Mn10_w sample, prepared from SnO2_Mn10 by etching with the concentrated mixture of H_2_C_2_O_4_ and HCl. It was supposed that manganese be removed from the surface of the nanocomposite as a soluble form of Mn (II)–oxalic complex or chloride salt. Still, manganese intruded into the SnO_2_ lattice is not, because of the high resistance of SnO_2_ to acids in opposite to excellent reactivity of any MnO_x_ with H_2_C_2_O_4_ or HCl. Therefore, manganese forms substitution defects in the SnO_2_ rutile-type lattice [8,11,12]. Furthermore, according to the known Shannon’s ionic radii values, the only manganese state possible to replace Sn (IV) in octahedral coordination (r_ionic_ = 69 pm) is the same coordinated high-spin Mn(III) ion (r_ionic_ = 64.5 pm). The EDX elemental composition data are presented in Table 4.

One of the main disadvantages of the EDX technique–a matrix effect–may bring an unavoidable systematic error in the quantitative analysis results. Total reflection X-ray fluorescence spectroscopy (TXRF) is considered to be a more accurate method for elemental analysis, to the same extent as atomic emission spectroscopy (AES) with inductively coupled plasma (ICP) [29]. So, the trial to verify EDX data and determine manganese surface/lattice concentrations was carried out using TXRF with internal standard (Co^2+^ solution).

The suspensions of SnO2-300, SnO2_Mn1 and SnO2_Mn10 were analyzed to determine the total manganese concentration. Surface-located manganese concentration was determined by examination of supernatant solutions of mixtures of the same samples with oxalic and hydrochloric acids. It was supposed to calculate the concentration of manganese intruded in the SnO_2_ crystal structure as the difference between total and surface manganese concentrations. Unfortunately, this technique appeared to be not accurate enough (Table 5) and gives inadequate absolute values for analysis of suspensions which gives the total manganese quantity in materials.

To solve this analytical problem, ICP MS was used to determine low concentrations of manganese in acid-etched samples SnO2_Mn1_w and SnO2_Mn10_w. (Table 6). The obtained values were accepted as the concentrations of manganese in the crystal structure of SnO_2_ in samples SnO2_Mn1 and SnO2_Mn10, respectively. Molar fractions of Sn and Mn were calculated assuming Mn and Sn in SnO2_Mn1_w and SnO2_Mn10_w as stoichiometric Mn_2_O_3_ and SnO_2_.

Therefore, the total concentration of manganese was calculated by summation lattice Mn concentration revealed by ICP MS and surface Mn content determined by TXRF. Assuming that tin presents in the samples as SnO_2_, lattice manganese as Mn_2_O_3_ and surface manganese as MnO_2,_ it became possible to calculate molar fractions of Sn and Mn in materials (Table 7).

The values indirectly calculated from TXRF and ICP MS molar ratio Mn: Sn converge with direct results of TXRF analysis of suspensions. EDX gives overestimated manganese concentrations, especially for the SnO2_Mn10_w sample. According to the ICP MS data, the manganese solubility limit in the SnO_2_ lattice is not reached for samples with Mn: Sn total ratio of less than 10 mol. % that means a possibility of material evolution with manganese surface-to-lattice migration during the continuous heating at 300 °C.

In summary, SnO_2_/MnO_x_ nanocomposites prepared by the impregnation-annealing technique are heterogeneous materials made of “multilayer particles”. The thin discontinuous top layer is composed of MnO_2_. The middle layer can be imagined as Mn (III)-rich defected SnO_2_ structure, and the inner one is probably SnO_2_ nearly free of Mn (III).

### 3.4. Investigation of Active Centers on the Surface of Blank SnO_2_ and SnO_2_/MnO_x_ Composites

**Figure 7:**
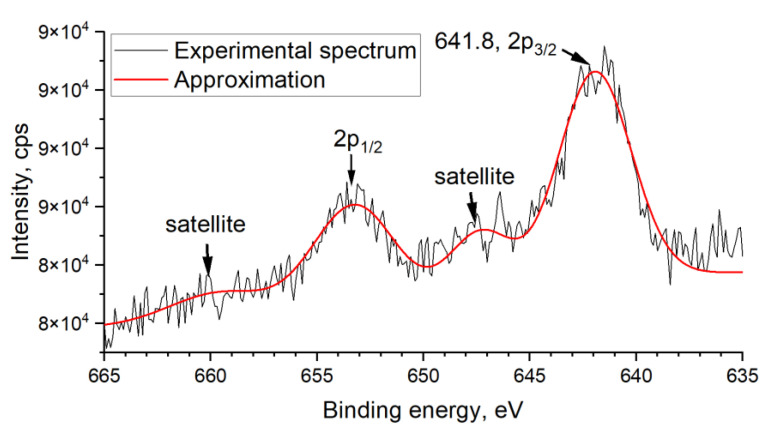
XPS spectrum of SnO2_Mn1 in Mn 2p region.

X-ray photoelectron spectroscopy (XPS) is one of the most advanced techniques for discovering the oxidation state of elements in different compounds. The influence of chemical factors on electron binding energy (BE) is the biggest for the pre-valence shell of the atom. Thus, using the Mn 3s region to determine the oxidation state is better. However, the attempt to obtain the Mn 3s spectrum for SnO2_Mn1 failed due to apparatus restrictions and too low manganese content. Besides observed Mn 2p spectrum was useless for this aim: although the obtained binding energy of 641.8 eV for Mn 2p_3/2_ can be attributed to Mn (IV) in MnO_2_ [30], according to the published data, the difference between Mn 2p_3/2_ peaks for Mn +3 and+4 consists only 0.5–1 eV [31,32]. This fact makes XPS analysis of the manganese chemical state uninformative for such samples as SnO2_Mn1 (Figure 7) or SnO2_Mn10_w, where Raman spectroscopy can’t detect the manganese-containing phase.

**Figure 8:**
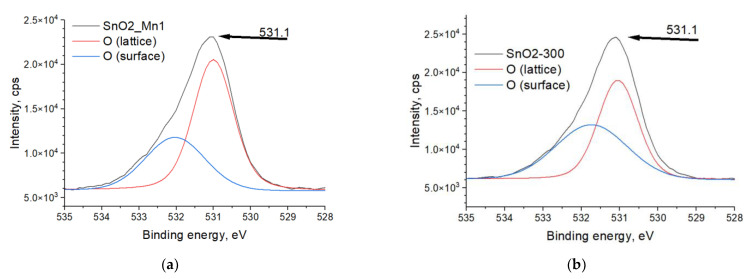
XPS spectra of (**a**) SnO2-300 and (**b**) SnO2_Mn1 in the O 1s region. The «O (lattice)» curve corresponds to the main peak, and «O (surface)»–is the minor peak.

As for oxide-based sensing materials, XPS is considered helpful in investigating the chemical state of oxygen, which gives O 1s spectra in the shape of one asymmetric broad peak (Figure 8a,b). There is no exact rule on how to fit this peak by Gauss-Lorentz functions, but in general, it is supposed that the main sharp peak corresponds to oxygen atoms of oxide structure, minor diffuse peak matches surface oxygen state like hydroxyl groups, adsorbed water, and chemisorbed oxygen [33,34]. The location of the O (lattice) peak is the same for both analyzed samples, although the ratio of minor and main peak areas differs (Table 8).

As shown in Table 8, impregnation-annealing modification of SnO_2_ by manganese results in a decrease in surface oxygen concentration compared to blank SnO_2_. This effect could be a consequence of manganese (III) low-valence doping of SnO_2_, which leads to the formation of an electron-depleted layer on the surface of the material and a decrease in its affinity to oxygen.

**Figure 9:**
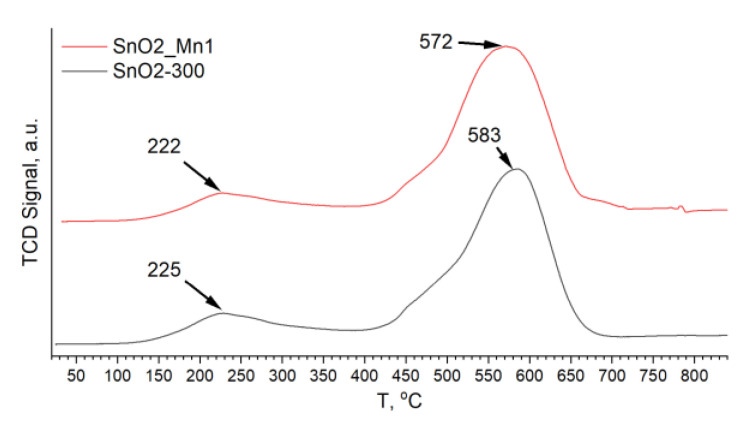
TPR-H_2_ curves of SnO2-300 and SnO2_Mn1.

Another helpful method is a thermo-programmed reduction with hydrogen (TPR-H_2_), which describes the change of the material’s redox properties and the quantity of chemisorbed oxygen [2]. There are two regions on the TPR-H_2_ curve of nanocrystalline SnO_2_-based materials (Figure 9): low-temperature (approx. 100–350 °C) and high-temperature. (approx. 350–750 °C). The area of the first peak is proportional to the quantity of chemisorbed oxygen. In contrast, the position of the peak maximum is related to the ratio of different chemisorbed oxygen forms: the lower the peak maximum temperature, the stronger the oxidizing agent prevails among the forms of chemisorbed oxygen.

The high-temperature peak corresponds to the reduction of tin dioxide to metal. The position of the maximum temperature of the peak is related to the oxidation potential of the material but may undergo a shift depending on the parameters of the microstructure. Modifying SnO_2_ with MnO_x_ leads to a shift of the high-temperature peak to lower temperatures by approx. 10 °C. This indicates the participation of the manganese-containing phase in the reduction of SnO_2_ with hydrogen. The differences in the position of the chemisorbed oxygen reduction peak between modified and unmodified SnO_2_ are much smaller. In this case, there is a significant difference in the ratios of the peak areas of the reduction of chemisorbed oxygen and oxide (Table 9).

The manganese-modified material has a smaller contribution from the reduction of chemisorbed oxygen to the uptake of hydrogen by the sample, which agrees with XPS results. The total amount of hydrogen consumed during the reduction of the samples is less than the theoretical value n(H_2_)/n(SnO_2_) = 2, corresponding to the reduction of stoichiometric SnO_2_ to Sn^0^. This may be due to the high defectiveness of tin dioxide obtained at a low annealing temperature of 300 °C.

The additional information about manganese and chemisorbed oxygen state was obtained by electron paramagnetic resonance spectroscopy (EPR). This method deals with paramagnetic centers only, which were presented in SnO_2_/MnO_x_ materials as manganese ions and oxygen-containing radicals.

**Figure 10:**
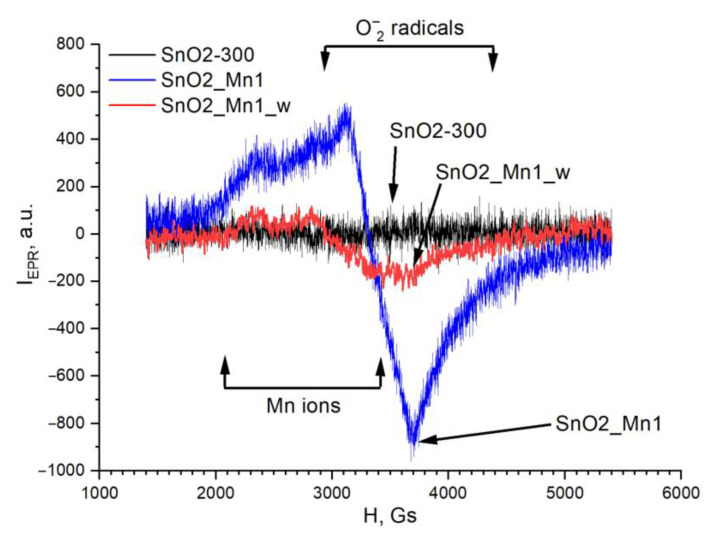
EPR spectra of SnO2-300, SnO2_Mn1 and SnO2_Mn1_w. Regions of signals of manganese ions and O_2_^−^ ions are highlighted.

The EPR spectrum of unmodified SnO_2_ does not demonstrate the signals related to superoxide radicals and manganese ions (Figure 10). The spectra of sample SnO2_Mn1 are a superposition of the EPR signals of manganese (III) ions (or manganese ions in other oxidation states) and oxygen radical anions O_2_^−^ (g_1_ = 2.02, g_2_ = 2.009, g_3_ = 2.003). The observed lines in the spectra are strongly broadened due to dipole-dipole and/or exchange interactions. Moreover, the hyperfine splitting caused by the interaction of an unpaired electron with the paramagnetic (I = 5/2) nucleus of the manganese atom contributes to the broadening of the EPR signal of manganese ions.

The EPR spectrum of the etched SnO2_Mn1_w sample discerns from the others by weaker signals from both manganese ions and O_2_^−^ radicals. The first observation is explained by a significantly lower (about three times) manganese content in the material compared to sample SnO2_Mn1. Second, because of the signal absence of oxygen anion-radicals O_2_^−^ in the spectrum of unmodified SnO_2_, one can conclude that manganese in the form of MnO_x_ segregation on the surface of SnO_2_ promotes the formation of superoxide ions. Manganese introduced into the crystal structure of SnO_2_ does not bring such an effect. This observation does not contradict the results of studying the state of oxygen by XPS since EPR is sensitive only to paramagnetic forms of oxygen. At the same time, XPS is equally sensitive to all.

The detection of O_2_^−^ ions in the EPR spectra obtained at room temperature agrees with the published data: superoxide ions are one of the forms of chemisorbed oxygen, which is most stable at temperatures up to 200 °C [1]. These particles are also responsible for forming the sensor response of the material toward reducing gases when operating in the designated temperature range.

### 3.5. Gas Sensing Properties of Nanocrystalline SnO_2_ and SnO_2_/MnO_x_ Nanocomposites toward CO and NO

**Figure 11:**
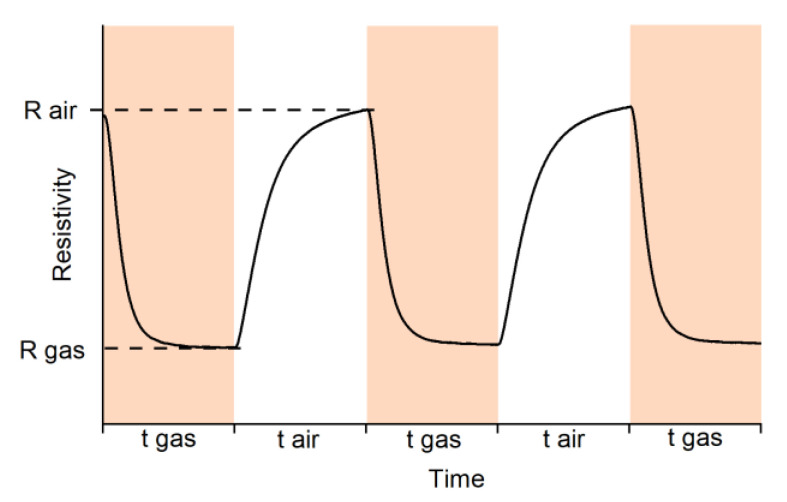
A typical shape of resistance dependence on test gas composition of n-type semiconducting oxide during the reducing gas detection.

When detecting CO, a decrease in the resistance of SnO2_300 and SnO2_Mn_1 was observed in the presence of the target gas, which is typical for n-type semiconductors (Figure 11). This decrease in resistance is due to the CO oxidation with chemisorbed oxygen:

(5) $$2{\mathrm{CO}}_{\left(\mathrm{gas}\right)}+O_{2\left(\mathrm{ads}\right)}^{-}\to 2{\mathrm{CO}}_{2\left(\mathrm{gas}\right)}+e^{-}$$

(6) $${\mathrm{CO}}_{\left(\mathrm{gas}\right)}+O_{\left(\mathrm{ads}\right)}^{-}\to {\mathrm{CO}}_{2\left(\mathrm{gas}\right)}+e^{-}$$

where CO_(gas)_ is CO molecule in the gas phase, $O_{2\left(\mathrm{ads}\right),}^{-}O_{\left(\mathrm{ads}\right)}^{-}$ are different forms of chemisorbed oxygen, CO_2(gas)_ is a product of the oxidation of CO gas desorbed into the gas phase. Upon change to an atmosphere of dry air, a return of the resistance to the value close to the initial value in the air was observed.

**Figure 12:**
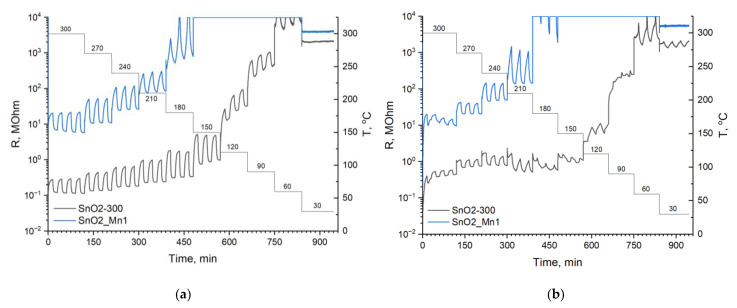
Resistance curves of SnO2-300 and SnO2_Mn1 during the cyclic test gas composition change at different temperatures when detecting: (**a**) 20 ppm CO; (**b**) 4 ppm NO.

The resistance of SnO2_Mn1 appeared to be higher than that of unmodified SnO_2_ by two orders of magnitude (Figure 12a). Previously, it was shown that manganese cations are introduced into the SnO_2_ crystal structure, and these observations indicate the formation of acceptor defects in the SnO_2_ structure in SnO_2_/MnO_x_ composites. Therefore, the oxidation state of manganese intruded SnO_2_ crystal structure is less than +4. Considering the ionic radii of the Mn^n+^ cations in an octahedral oxygen environment and the stability diagram of manganese oxides [35], the most suitable manganese cation for the SnO_2_ structure is the high-spin Mn^3+^, and the reaction of formation of a solid solution in SnO_2_ will take the following form (Kröger–Vink notation):

(7) $${\mathrm{Mn}}_{2}O_{3}\overset{2{\mathrm{SnO}}_{2}}{\to }2{\mathrm{Mn}}_{\mathrm{Sn}}^{\prime }+3O_{O}^{\times }+V_{O}$$

It is shown in Figure 12 that the resistance of materials increases with decreasing temperature. However, for temperatures below 180 °C, a correct resistance measurement for the SnO2_Mn1 material is impossible because of overcoming the threshold value of used equipment.

Measurements in NO containing atmosphere (4 ppm NO in dry air) revealed that the SnO_2_ signal shape has a more complex profile (Figure 12b) than a typical one (Figure 11). In this case, signal inversion is observed for tin dioxide at temperatures below 150 °C.

During the interaction of SnO_2_ and SnO_2_/MnO_x_ composites with NO, several competing reactions can occur with the capture of electrons from the conduction band of SnO_2_:

(8) $${\mathrm{NO}}_{\left(\mathrm{gas}\right)}+\frac{1}{2}O_{2\left(\mathrm{gas}\right)}\to {\mathrm{NO}}_{2\left(\mathrm{gas}\right)}$$

(9) $${\mathrm{NO}}_{2\left(\mathrm{gas}\right)}+e^{-}\to {\mathrm{NO}}_{2\left(\mathrm{ads}\right)}^{-}$$

and vice versa, with the injection of electrons localized on chemisorbed oxygen into the conduction band of SnO_2_:

(10) $${\mathrm{NO}}_{\left(\mathrm{gas}\right)}+O_{2\left(\mathrm{ads}\right)}^{-}\to {\mathrm{NO}}_{2\left(\mathrm{gas}\right)}+\frac{1}{2}O_{2\left(\mathrm{gas}\right)}+e^{-}$$

The modification of SnO_2_ with manganese oxides leads to the prevalence of the NO oxidation reaction with the participation of ionized forms of chemisorbed oxygen, which agrees with the results obtained by the EPR spectroscopy.

The likely reason for the inversion of the SnO_2_ signal when detecting NO is a decrease in the concentration of chemisorbed oxygen forms with strong oxidizing properties. As shown in previous works, at temperatures below 200 °C, physically adsorbed O_2_ molecules and O_2_^−^ radical anions predominate on the SnO_2_ surface. At the same time, tin dioxide, synthesized in this work, is characterized by an extremely low concentration of chemisorbed oxygen in O_2_^−^ form (Figure 10). According to the Weitz limitation [36], in the case of equilibrium ionosorption, the degree of surface coverage by charged particles cannot exceed 10^−3^ parts of the monolayer. Thus, an increase in the concentration of chemisorbed oxygen in O_2_^−^ form upon modification of the SnO_2_ surface with manganese oxides (Figure 10) hinders the adsorption of the resulting NO_2_ in NO_2_^−^ form (reaction (9)), which eliminates the inversion of the sensor response.

**Figure 13:**
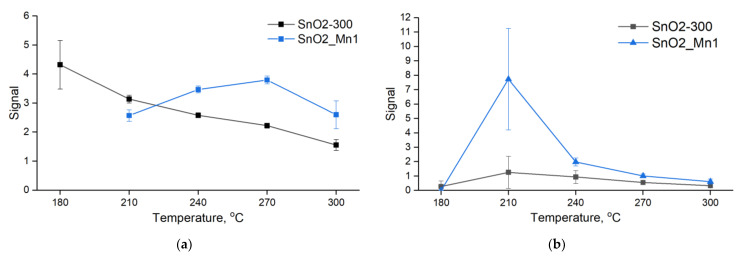
Temperature dependence signal curves of SnO2-300 and SnO2_Mn1 when detecting: (**a**) 20 ppm CO; (**b**) 4 ppm NO.

The temperature dependences of the sensor response of SnO_2_ and SnO2_Mn1 materials toward CO and NO are shown in Figure 13a,b. High values of the standard deviation of the signal magnitude at low temperatures may be evidence of a violation of the reversibility of the reactions that determine the sensor response of the material and may be a consequence of hardware noise when measuring high resistances.

To study the influence of the processes leading to the formation of a sensor response, the concentration dependences of the signal when detecting CO and NO at temperatures of the maximum stable signal was investigated. In addition, to clarify the effect of MnO_x_ surface segregation on the sensor properties of SnO_2_, a sensor was made of SnO2_Mn1_w material, which contains manganese only in the form of cations introduced into the SnO_2_ structure.

**Figure 14:**
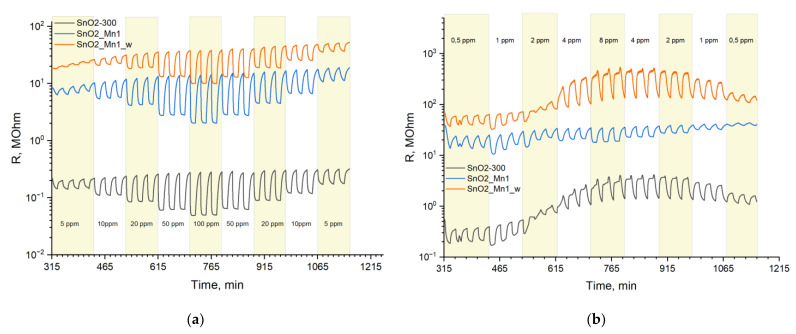
Resistance curves of SnO2-300, SnO2_Mn1 and SnO2_Mn1_w during the cyclic test gas composition change at different gas concentrations to detect: (**a**) CO; (**b**) NO at 270 °C.

The signal of SnO2-300 and SnO_2_/MnO_x_ composites toward CO seemed to be the simplest and most predictable. For this reason, CO was chosen to study general patterns. The dependences of the materials resistance with a change in CO concentration at constant measurement temperature are shown in Figure 14a.

The resistance of the SnO2_Mn1_w material was found to be on par with the parent SnO2_Mn1 material. Therefore, an increase in the resistance of tin dioxide modified with manganese oxides is a consequence of the incorporation of manganese (III) cations into the crystal structure of the oxide (Equation (7)).

**Figure 15:**
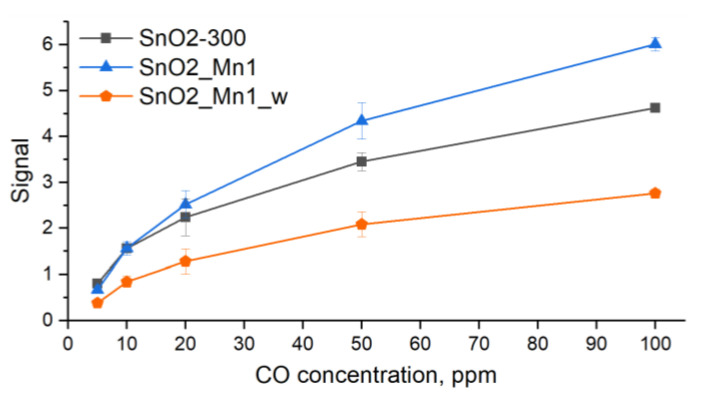
During the cyclic test gas composition, the signal concentration-dependence curves of SnO2-300, SnO2_Mn1 and SnO2_Mn1_w change to detect CO descending from 100 ppm to 5 ppm.

Even after prolonged annealing, as seen in Figure 14a, there is some drift of the baseline (resistance in clean air), which indicates that the equilibrium was not fully reached during the measurements. The signal value of the SnO2_Mn1 material is slightly superior compared to unmodified SnO2-300, while the SnO2_Mn1_w material is noticeably inferior to SnO_2_ (Figure 15).

When studying the temperature dependence of the signal to nitrogen monoxide, it was found that unmodified SnO_2_ in a certain temperature range demonstrate signal inversion. To explain the observed phenomena, NO was chosen to study the concentration dependence of the signal (Figure 14b). The results show that the sensor signal of etched SnO2_Mn1_w has the same shape as that of SnO2-300. Therefore, the surface segregation of MnO_x_ defines the shape of the sensor signal of the SnO_2_/MnO_x_ composites toward NO.

**Figure 16:**
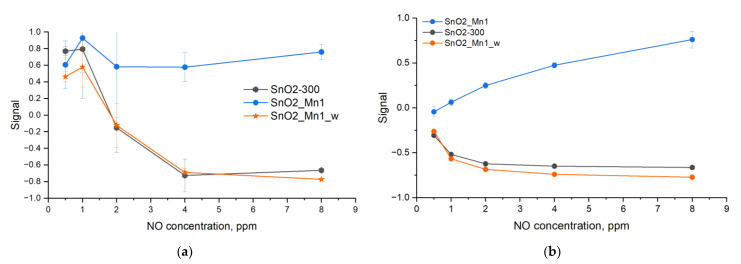
Signal concentration-dependence curves of SnO2-300, SnO2_Mn1 and SnO2_Mn1_w during the cyclic test gas composition change when detecting NO at 270 °C: (**a**) ascending from concentration from 0.5 to 8 ppm, (**b**) descending concentration from 8 to 0.5 ppm.

Note that the signal decreases for SnO2_Mn1 as the NO concentration increases from 1 ppm to 2 ppm (Figure 16a). Unmodified SnO2-300 and SnO2_Mn1_w in this interval detects NO as a reducing agent and, when the concentration exceeds 4 ppm, even when descending gas concentration, as an oxidizing agent (Figure 16b). It means that the action of sufficiently high concentrations of nitrogen monoxide causes poisoning of the surface of the material by NO oxidation products according to Reaction (9), causing the replacement of chemisorbed oxygen by surface nitrite ions, which leads to the suppression of Reaction (10). The presence of MnO_x_ segregation on the SnO_2_ surface does not exclude the poisoning of the material surface. Still, it promotes Reaction (10), as a result of which the SnO2_Mn1 material has a time-stable signal toward NO and a small baseline drift (Figure 16b).

## 4. Conclusions

Nanocomposites SnO_2_/MnO_x_ with 4–5 nm grain size and specific surface area of 100–105 m^2^/g were prepared by the chemical precipitation of SnO_2_ and its modification with MnO_x_ by impregnation-annealing technique. The detailed characterization was carried out to determine microstructure parameters (SEM, XRD, BET, Raman spectroscopy), to investigate phase and elemental composition of synthesized materials, including manganese distribution between bulk and surface of SnO_2_ grains (Raman spectroscopy, EDX mapping, TXRF, ICP-MS) and, finally, to obtain the information about the chemical state of active surface centers (XPS, EPR, TPR-H_2_). Several significant observations were made: (1) although the manganese is introduced as a surface modifier, there is a doping effect caused by the redistribution of manganese between surface segregation and SnO_2_ crystal structure; (2) manganese presents in SnO_2_/MnO_x_ nanocomposites in two different states–as MnO_2_ surface phase and as Mn(III) ions in the SnO_2_ crystal structure; (3) surface manganese-containing segregation has a significant influence on sensor properties of SnO_2_/MnO_x_ nanocomposites. The presence of manganese in the total concentration of Mn: Sn = 1 mol.% increases SnO_2_ sensitivity when detecting typical reducing gas CO and prevents signal inversion when detecting NO.

## Figures and Tables

**Table 1:** Samples designations and synthesis conditions.

Sample Name	Preassigned Mn:Sn Ratio, mol. %	Prepared from	Acid Etching
SnO2-300	0	-	No
SnO2_Mn1	1%	SnO2-300	No
SnO2_Mn10	10%	SnO2-300	No
SnO2_Mn1_w	-	SnO2_Mn1	Yes
SnO2_Mn10_w	-	SnO2_Mn10	Yes

**Table 2:** The phase composition and microstructure parameters of prepared SnO_2_ and SnO_2_/MnO_x_ nanocomposites.

Sample	Phase Composition by XRD	d_XRD_, nm	S_surf_, m^2^/g	d_BET_, nm	d_SEM_, nm
SnO2-300	SnO_2_	3.5–4.0	120	7	5–10
SnO2_Mn1	SnO_2_	4.0–4.5	105	8	5–10
SnO2_Mn10	SnO_2_	4.5–5.0	100	8.5	5–10
SnO2_Mn1_w	SnO_2_	4.0–4.5	105	8	5–10
SnO2_Mn10_w	SnO_2_	4.0–4.5	-	-	5–10

**Table 3:** Colors of samples and properties of the peaks of Raman spectra obtained with a 633 nm radiation source. The peak at 628 cm^−1^ is named «sharp», and the wide band at approx. 553 cm^−1^ named «broad». I_sharp_ means the intensity of a sharp peak, and FWHM_sharp_ is full width at half maximum for a sharp peak.

Sample	Color	Raman Shift_sharp_	Sharp/S_broad_	I_sharp_/FWHM_sharp_
SnO2-300	Creamy	632	0.09	1.1
SnO2_Mn1	Sand	630	0.06	2.0
SnO2_Mn1_w	Sand	630	0.10	2.3
SnO2_Mn10	Black	630	1.8	0.65
SnO2_Mn10_w	Sand	630	0.08	2.2
MnO_2_	Black	633	2.2	1.0

**Table 4:** Elemental composition (in molar fractions) of SnO_2_/MnO_x_ materials obtained by EDX.

Element	SnO2_Mn1	SnO2_Mn10	SnO2_Mn10_w
Sn	0.99	0.88	0.99
Mn	0.01	0.12	0.01

**Table 5:** Elemental composition of SnO_2_/MnO_x_ materials obtained by TXRF. Given errors were calculated from known method errors.

Element	SnO2-300	SnO2_Mn1	SnO2_Mn10
Sn (molar fraction)	1	0.99 ± 0.004	0.89 ± 0.04
Mn (total, molar fraction)	0	0.01 ± 0.004	0.11 ± 0.04
Mn (total, weight fraction)	0	1.4·10^−3^ ± 0.6·10^−3^	20·10^−3^ ± 8·10^−3^
Mn (surface, weight fraction)	0	1.4·10^−3^ ± 0.2·10^−3^	31·10^−3^ ± 6·10^−3^

**Table 6:** The manganese content in blank SnO_2_ and etched SnO_2_/MnO_x_ materials obtained by ICP MS. Given errors were calculated from known method errors.

Element	SnO2-300	SnO2_Mn1_w	SnO2_Mn10_w
Mn (total, weight fraction)	1·10^−5^ ± 0.05·10^−5^	90·10^−5^ ± 4.5·10^−5^	200·10^−5^ ± 10·10^−5^
Sn (molar fraction)	1.0	0.9975 ± 0.0001	0.9945 ± 0.0003
Mn (total, molar fraction)	0.0	0.0025 ± 0.0001	0.0055 ± 0.0003

**Table 7:** The manganese content in SnO_2_/MnO_x_ materials obtained by TXRF and ICP MS. Given errors were calculated from known method errors.

Element Quantity	SnO2_Mn1	SnO2_Mn10
Mn (total, weight fraction)	2.3·10^−3^ ± 0.245·10^−3^	33·10^−3^ ± 6.1·10^−3^
Mn (surface, weight fraction)	1.4·10^−3^ ± 0.2·10^−3^	31·10^−3^ ± 6·10^−3^
Mn (lattice, weight fraction)	0.9·10^−3^ ± 0.045·10^−3^	2·10^−3^ ± 0.1·10^−3^
Mn (surface)/Mn (total)	0.6 ± 0.15	0.94 ± 0.25
Sn (molar fraction)	0.994 ± 0.9·10^−3^	0.91 ± 14·10^−3^
Mn (total, molar fraction)	0.006 ± 0.9·10^−3^	0.09 ± 14·10^−3^
Mn (surface, molar fraction)	0.0035 ± 0.4·10^−3^	0.0845 ± 8·10^−3^
Mn (lattice, molar fraction)	0.0025 ± 0.4·10^−3^	0.0055 ± 0.8·10^−3^

**Table 8:** XPS data for Sn 3d, Mn 2p and O 1s regions. S_rel_–areas of oxygen main (lattice) and minor (surface) peaks related to the total peak area.

Sample	BE Sn 3d_5/2_, eV	BE Mn 2p_3/2_, eV	BE O 1s, eV	S_rel_(O_lattice_)	S_rel_(O_surface)_
SnO2-300	487.1	-	531.1	0.52	0.48
SnO2_Mn1	487.2	641.8	531.1	0.64	0.36

**Table 9:** TPR-H_2_ data for SnO2-300 and SnO2_Mn1. T_max_1—temperature of low-temperature peak maximum, T_max_2—temperature of high-temperature peak maximum; S_2_, S_1_ and S_total_ are areas of high-, low-temperature peaks and their sum, respectively; n(H_2_)/n(SnO_2_)—quantity of H_2_ consumed during reduction of 1 mole of SnO_2_.

Sample	T_max_1, °C	T_max_2, °C	S_1_/S_2_	S_1_/S_total_	n(H_2_)/n(SnO_2_)
SnO2-300	225	583	0.2	0.16	1.8
SnO2_Mn1	222	572	0.16	0.14	1.9

## Data Availability

The data that support the findings of this study are available from the corresponding author upon reasonable request.

## Supplementary Materials

The following are available online at https://www.mdpi.com/article/10.3390/nano13091437/s1, Figure S1: Powder X-ray diffraction pattern of synthesized Mn(acac)_3_ in comparison with a calculated pattern of δ-Mn(acac)_3_; Figure S2: SEM images obtained with Zeiss Supra 40 FE-SEM microscope at ×750·10^3^ of the following samples: (a) SnO2_Mn10; (b) SnO2_Mn1_w; Figure S3: SEM images, Sn and Mn EDX maps of SnO2_Mn1 (a–c), SnO2_Mn10 (d–f), SnO2_Mn10_w (g–i), respectively, at ×10^5^ magnification.
