# Supplementary material for: Effect of Manganese Distribution on Sensor Properties of SnO2/MnOx Nanocomposites
Source: Nanomaterials (Basel). 2023 Apr 22;13(9):1437. doi: 10.3390/nano13091437 (PMC10179853; doi:10.3390/nano13091437)
Supplement: Supplementary file 1 [file nanomaterials-13-01437-s001.zip › nanomaterials-2345851-supplementary.pdf]

Effect of Manganese Distribution on Sensor Properties of SnO_2/MnO_x Nanocomposites

Rodion Eshmakov [1], Darya Filatova [1], Elizaveta Konstantinova [2], and Marina Rumyantseva [1]

[1] Chemistry Department, Moscow State University, 119991 Moscow, Russia
[2] Physics Department, Moscow State University, 119991 Moscow, Russia

Supplementary Information

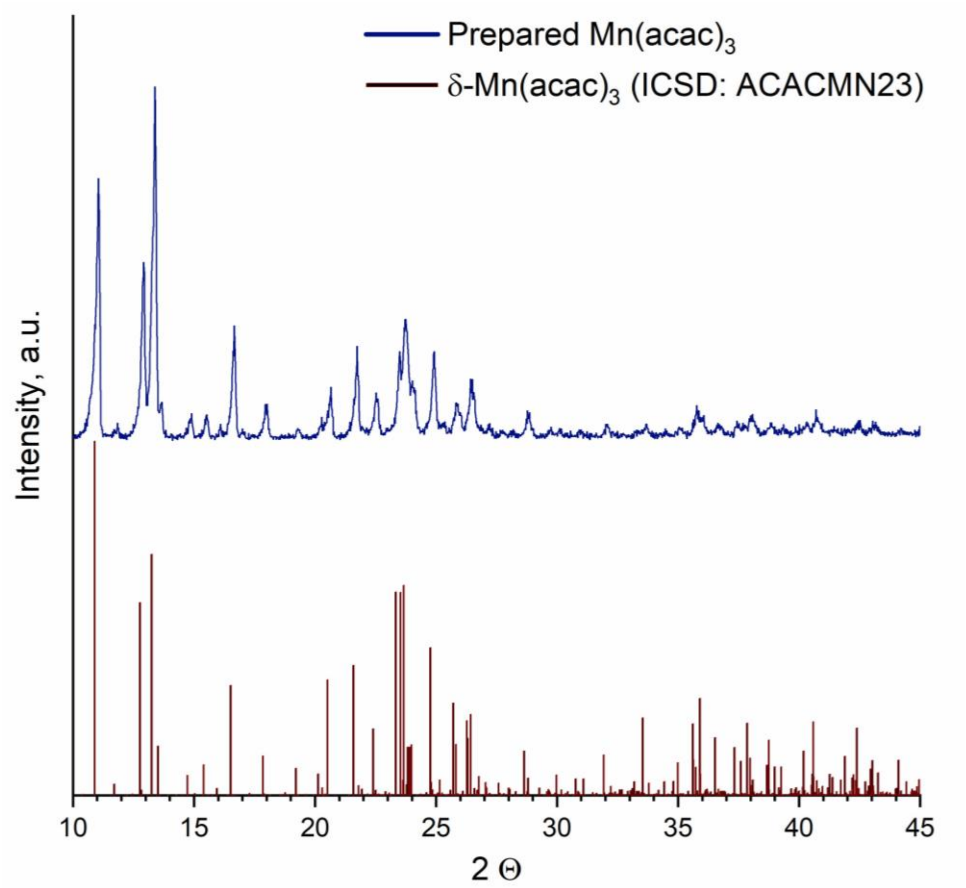

Figure S1. Powder X-ray diffraction pattern of synthesized $Mn(acac)_3$ in comparison with calculated pattern of δ-$Mn(acac)_3$.

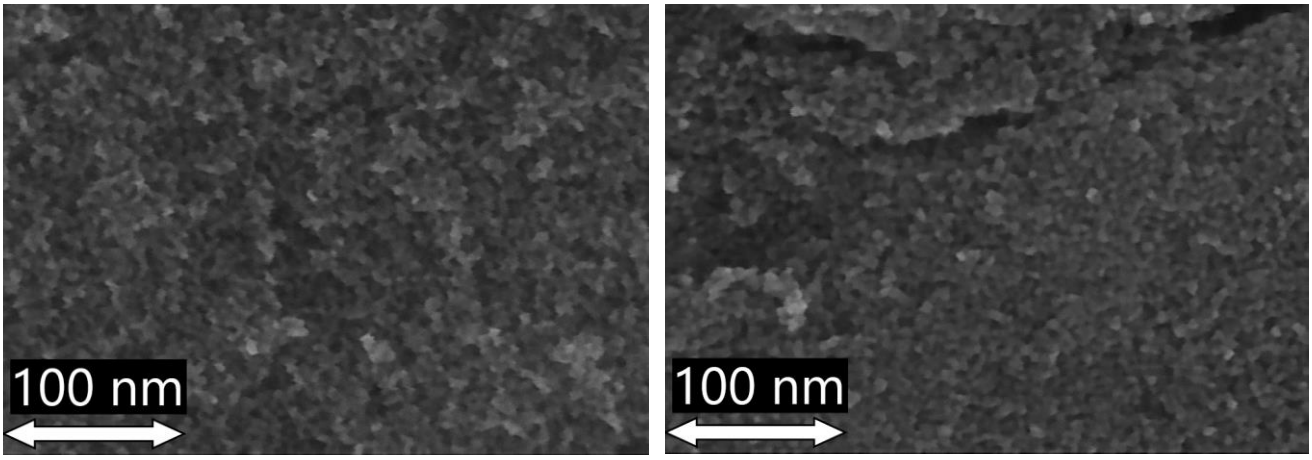

(a) **(b)**

Figure S2. SEM images obtained with Zeiss Supra 40 FE-SEM microscope at ×750·10^3 of the following samples: (**a**) SnO2_Mn10; (**b**) SnO2_Mn1_w.

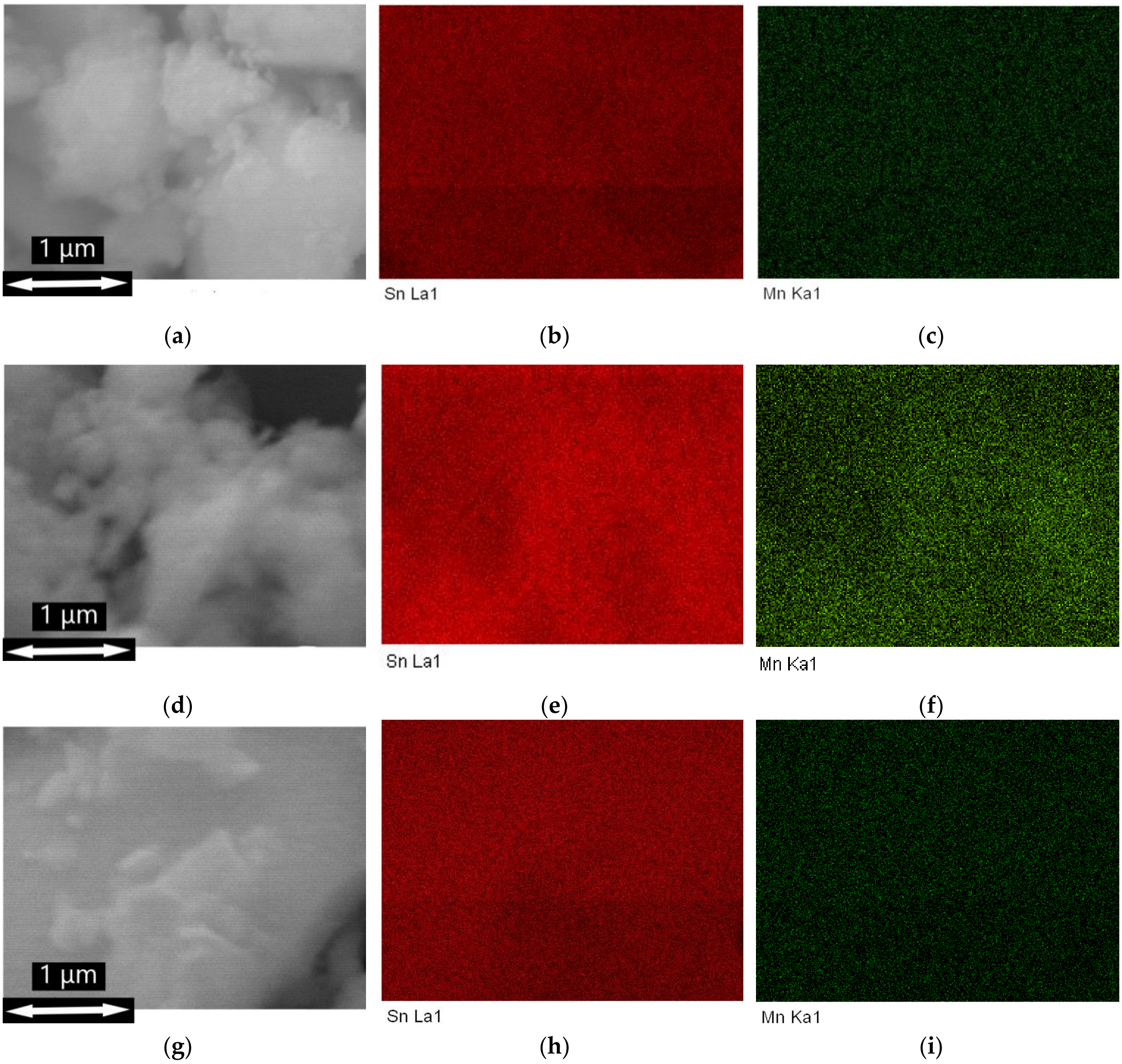

Figure S3. SEM images, Sn and Mn EDX maps of SnO2_Mn1 (**a-c**), SnO2_Mn10 (**d-f**), SnO2_Mn10_w (**g-i**), respectively, at ×10^5 magnification.
